# Supplementary material for: The influence of saccades on yaw gaze stabilization in fly flight
Source: PLoS Comput Biol. 2023 Dec 21;19(12):e1011746. doi: 10.1371/journal.pcbi.1011746 (PMC10769041; doi:10.1371/journal.pcbi.1011746)
Supplement: Attachment — Submitted filename: Response_v1.docx [file pcbi.1011746.s001.docx]

We thank both reviewers for their constructive feedback. Below we provide a point-by-point response. We also include references at the end.

**Reviewer #1**

*This study seeks to extend a model the influence of saccades on yaw gaze stabilization in fly flight. In this research article, the authors extend our understanding of the benefits of combining smooth and saccadic movements in the fruit fly D. melanogaster to improve flight stability and performance.*

*This study formulates a hybrid control system model that incorporates continuous (smooth) and discrete (saccadic) wing steering movements to stabilize visual gaze against perturbations to optic flow, or the pattern of motion cues projected onto the eyes as the fly flies through a stationary visual landscape. Prior work had done something similar, but without considering the biomechanical constraints of the motor/mechanical locomotory plant. Furthermore, prior work had modeled visual gaze stabilization with, effectively, a simultaneous hybrid controller, whereas here the authors formally consider a switched hybrid controller. The distinction is nontrivial as the two different hybrid control systems involve both continuous and discrete dynamics, but the implications to underlying neural circuit mechanisms are vastly different (and ought to be touched upon in revision).*

*Prior work by the senior author focusing on saccadic object tracking behavior developed a switched hybrid system that integrates object position error and background velocity error for object tracking and yaw gaze stabilization. Here, the authors more fully explore a model for smooth and saccadic responses that stabilize visual gaze against continuous wide-field optic flow. The model uses state vector transitions according to a flow map, and transitions discontinuously according to a jump map.*

*The hybrid control system model contains gain, delay, and switching threshold parameters that would contribute to the optomotor yaw stabilization reflex of the fruit fly D. melanogaster. The model includes a first-order plant, a Proportional-Integral (PI) continuous controller, and a saccadic reset system that fires based on the integrated error of the continuous controller. The gain parameter determines the strength of the feedback loop, the delay parameter determines the time delay between the input and output signals, and the switching threshold parameter determines the threshold at which the saccadic reset system is activated.*

*The study systematically explores the gain, delay, and switching threshold parameter space to quantify the optimum regions for yaw stability and performance. The results show that the addition of saccades to a continuous controller provides benefits to both stability and performance across a range of visual motion speeds. A comprehensive parameter exploration of the model suggests that Drosophila operates near its optimal switching threshold between smooth and saccadic steering movements for its experimental gain set. Based on experimental data, D. melanogaster operates in a region that trades off performance and stability, which increases flight robustness and facilitates compensation for internal perturbations such as wing damage.*

*The work has several implications for bio-inspired control systems and the design of autonomous flying robots. First, the study shows that the combination of continuous and discrete movements can improve flight stability and performance, particularly at high frequencies or speeds. This finding could inspire the development of bio-inspired control systems that incorporate both types of movements to improve the performance of autonomous flying robots. Second, the study demonstrates the advantages of hybrid control systems, which can expand the stable gain space and provide robustness considerations for handling different kinds of input signals, such as in conditions in which an animal needs to modulate optomotor gain such as when encountering an odor plume. These advantages could be applied to general engineering control systems beyond the behavior of D. melanogaster. Finally, the study highlights the importance of considering trade-offs between performance and stability in the design of bio-inspired control systems and autonomous flying robots. By balancing these factors, it may be possible to improve the robustness and compensation capabilities of these systems. These implications are discussed in more detail by the authors.*

*There are several limitations to the model and results presented in this study, which are detailed appropriately by the authors. First, the saccadic system was simplified as a simple controller and plant reset. In reality, saccades are highly complex, feedforward and feedback-based phenomena that propagate through the plant. Therefore, future work should include a more detailed model of the saccade dynamics. Second, the parameter space was not completely explored in this study. Specifically, the study limited the parameter space by fixing t_d at a fly-relevant 20 ms and choosing a subset of values for the other parameters. Therefore, further exploration of the parameter space could provide additional insights into the behavior of the hybrid control system. Finally, the study only considered velocity control and did not weigh position control priorities, which would be important when the fly encountered visual objects such as landscape features. These limitations are discussed in detail.*

*Overall I find the work very interesting, highly rigorous, and timely. This work significantly extends prior models by M.Land (and others) that incorporated relevant visual processing signals, but did not consider constraints based on system biomechanics, nor considered state space transitions. This study will certainly influence future modeling efforts that incorporate underlying neural dynamics. I would endorse publication and have few substantive comments.*

**Response:** We thank the reviewer for the constructive feedback.

*Comments:*

*The authors formalize a switched hybrid controller, which implies parallel yet interacting neural circuitry for the control of smooth optomotor responses and saccadic body reorientations. I would urge the authors to consider the published evidence for the latter both in the Introduction and the Discussion to bolster their case.*

**Response:** We agree.

**Action Taken:** We now cite and discuss work that provides further behavioral and neural evidence for parallel control within the Introduction and Discussion, including:

Aptekar, J. W., Shoemaker, P. A., Frye, M. A. (2012). Figure tracking by flies is supported by parallel visual streams. Current Biology, 22(6), 482–487.

Frighetto, G., Frye, M. A. (2023). Columnar neurons support saccadic bar tracking in Drosophila. eLife, 12.

Salem, W., Cellini, B., Frye, M. A., Mongeau, J.-M. (2020). Fly eyes are not still: a motion illusion in Drosophila flight supports parallel visual processing. Journal of Experimental Biology, 223(10).

Egelhaaf, M. (1987). Dynamic properties of two control systems underlying visually guided turning in house-flies. Journal of Comparative Physiology A, 161(6), 777–783.

Schnell, B., Ros, I. G., &#38; Dickinson, M. H. (2017). A Descending Neuron Correlated with the Rapid Steering Maneuvers of Flying Drosophila. Current Biology, 27(8), 1200–1205.

*An advantage of hybrid control could be discussed is that the biological gain set is modulated by cross-modal behaviors such as odor plume tracking, in which experimental evidence shows that continuous gain is increased, or possibly internal state, in which hunger or thirst modulates search or stability algorithms or heuristics.*

**Response:** We assume that the reviewer is referring to instances where visuomotor response gains are enhanced by the presence of a non-visual cue, such as an attractive odor (e.g., work by the Frye lab and others that olfactory input can gate visuomotor responses). For instance, active plume tracking of an attractive odor decreases saccade amplitude and frequency (Duistermars et al., 2009; Wasserman et al., 2012) and increases the gain of the yaw optomotor response (Chow et al., 2011). These results suggest distinct modulations to these two control systems.

**Action taken:** None as we feel that tying our current work to multisensory integration is a bit speculative as, to our knowledge, it remains unclear how cross-modal behaviors would benefit from a hybrid (switched) control strategy, specifically in terms of gaze stability and performance. Furthermore, in a recent review paper (Cellini and Mongeau, 2020), we highlighted the potential advantages of a hybrid fixate-and-saccade strategy with respect to multisensory integration.

*Minor:*

*L49: change to object*

**Response:** Changed to “object”.

*L26: change to slip*

**Response:** Changed to “slip”.

*L56: “motivations” seems odd here, change to “advantages”?*

**Response:** Changed to “advantages”.

*L64: consider changing to: the continuous feedback controller is inhibited by efference copy*

**Response:** Added “…is inhibited—analogous to an efference copy—…”

*L199: change to improved*

**Response:** Changed to “improve”.

*L82: regarding this citation - how could this include phase lag due to inertia if these flies were rigidly tethered? Maybe you meant to cite a different paper?*

**Response:** We agree that this citation is not appropriate. Instead, we now cite the following work:

Salem, W., Cellini, B., Jaworski, E., Mongeau, J.-M. (2023). Flies adaptively control flight to compensate for added inertia. Proceedings of the Royal Society B: Biological Sciences, 290(2008), 2022.12.15.520540.

*Figure 2: define abbreviations in the Legend, such as “M. Stable”*

**Response:** Defined in caption.

**Reviewer #2**

*Drosophila has been widely used as a model system to study yaw optomotor responses used for stabilizing gaze and forward trajectories in tethered preparations. It has been shown that under these conditions, flies use both smooth following movements as well as saccades to stabilize moving patterns. In this paper, Brock and Mongeau present a hybrid control model for yaw gaze stabilization in Drosophila, which can switch from a continuous proportional-integral control system to a saccadic system, when the error exceeds a treshold. They then explore the parameter space with respect to stability and performance of the system. The hybrid system performs better than a purely continuous system only at high input frequencies and at low gain regimes, in which they claim Drosophila operates. They therefore conclude that flies trade-off performance for stability, which could make the system more robust.*

*I generally find the paper well written and the findings interesting and a useful contribution to the field. However, I generally think that the paper could be improved by a more in depth discussion as will be discussed in the points below.*

**Response:** We thank the reviewer for the constructive feedback.

*1. The conclusion regarding the trade-off between stability and robustness rests on the experimental observation of the relatively low gain parameters, which have been measured in a restricted preparation. How well established are these parameters? How might their estimate be affected by the conditions under which they have been obtained? How variable are they and is it possible that flies can adjust the gains depending on the situation? I think these are questions that should be discussed before drawing any conclusions based on these parameters.*

**Response:** The gain parameters are derived from several studies that focused on characterizing the yaw optomotor response using system identification in a yaw-free magnetic tether, which we have cited throughout. In one paper we showed that the *Drosophila* yaw optomotor response operates with a gain near unity for continuous visual rotation (Mongeau and Frye, 2017), which corroborated studies in free flight (Mronz and Lehmann, 2008), thus providing some assurance that the yaw optomotor response gain is not fundamentally altered by the constraints of the magnetic tether. More recently, we showed that frictional torque due to the magnetic tether is about two orders of magnitude lower than turning torques in flight (Cellini et al., 2022), which provides further assurance that the gain magnitude in the magnetic tether is naturalistic for yaw turns. Collectively, this suggests that the yaw-based optomotor response in the magnetic tether resembles smooth yaw turns in free flight. Indeed, flies can generate nearly pure yaw turns in free flight with little roll and pitch (Bergou et al., 2010).

Our estimate of the proportional and integral gains is derived from several studies by our group (Cellini et al., 2022; Salem et al., 2022; Salem et al., 2023) with a LTI control theoretic framework. In short, we found that when flies operate local to an equilibrium point (zero retinal slip), the yaw optomotor response is well captured by a LTI model, which models ~90% of the variance in response gain and phase. Our estimate of the gain set used in our manuscript is based on fitting transfer functions to gain and phase averages computed across many flies. We are not aware of other studies which have quantified closed-loop P&I yaw-based visuomotor gains using a frequency domain analysis (although there have been previous efforts at system modeling of visual flight speed control, which is a different context; (Fuller et al., 2014; Rohrseitz and Fry, 2011)).

In recent work, we have showed that the yaw optomotor response gain can be modified to compensate for wing damage and added inertia, which appears to be complemented by active damping from proprioceptive feedback (halteres) (Salem et al., 2022; Salem et al., 2023), which is line with previous studies of multisensory integration in *Drosophila* (Fuller et al., 2014). Further, proprioceptive feedback appears to directly modulate optomotor response gains via active damping of head movements (Cellini and Mongeau, 2022). Additionally, it is well known that an odor can modulate the gain of the yaw optomotor response at both behavioral and physiological levels (Chow and Frye, 2008; Chow et al., 2011; Wasserman et al., 2015). Thus, we absolutely agree with the reviewer that optomotor gains can be modulated, but flies appear to do this while concomitantly modulating active damping, which appears to serve to maintain stability (Cellini and Mongeau, 2022; Elzinga et al., 2012; Fuller et al., 2014; Salem et al., 2022; Salem et al., 2023). Indeed, it has been argued that active damping can alleviate the influence of visuomotor delay and allow visuomotor loops to operate at high gain while maintaining stability (Dickinson and Muijres, 2016).

**Action taken**: We now provide more context on the estimate of the gain set in the Results (first paragraph). We now state explicitly that the gain set (P & I) is based on transfer function fitting to gain and phase averaged across many flies using frequency domain system identification. In the last paragraph of the Discussion, we now note that flies can modulate gain based on context as a caveat.

*2. Does the model make predictions that could be tested experimentally? E.g. one might expect lower saccade rates at low frequency inputs and higher saccade rates at higher ones. Has something like that be observed? Since optomotor behavior has been so well described, a more in depth discussion of how the model aligns with previous findings would be useful.*

**Response:** For continuous visual rotation, flies operate with a gain near unity across a range of stimulus rotation speeds (Mongeau and Frye, 2017; Mronz and Lehmann, 2008), thus we would expect a small influence of stimulus rotation speed on saccade rate in closed-loop (yaw free or free flight). We previously showed a strong influence of visual motion speed on head saccade rate (which are linked to $\Delta$WBA hitches), but these measurements were made in open-loop (body-fixed tether) which pushed performance to extremes, i.e., extreme visual retinal slip due to saturation nonlinearities of the head (Cellini et al., 2021). To our knowledge, the influence of visual motion frequency on saccade rate has not been explicitly determined in free flight or a yaw-free tether. Thus, “optomotor saccades” may serve to reset gaze when pushed to large visual error, as argued in Cellini et al., 2020, 2021.

**Action taken:** We have added a new paragraph to the Discussion which describes experimental predictions and better contextualizes our work given previous findings.

*Minor:*

*Fig. 3 legend: Should this be citation 19 here for the gain parameters?*

**Response:** The citation Salem et al., 2022 is correct because it explicitly determined P & I parameters from transfer function fitting, whereas Cellini et al., 2022 only computed P (though the P gains are identical in both studies because integral control captures little of the variance, as demonstrated in Salem et al., 2022)

*The 3D plots (e.g. in Fig. 7) are hard to interpret. Maybe showing them from a different angle might help, so that the important points are easier to see.*

**Response:** Showing an isometric projection was a comprise to simultaneously visualize the influence of frequency and threshold on SAE.

*Line 169: It is not immediately clear, what the “shaded regions” are supposed to be in the plot.*

**Response:** We replaced to “solid shaded” to avoid confusion.

*Line 199: “improved performance”*

**Response:** Corrected.

*Line 212: It is still a bit puzzling to me, why the system would not operate at higher proportional gains. Wouldn’t the risk of instability be mitigated by having a hybrid control system as argued in the paragraph below?*

**Response:** We assume that when referring to “the system”, the reviewer is referring to flies. This is an interesting point. One possibility, as we have argued in this manuscript, is that a lower proportional gain provides flies with larger stability margins (and thus is more robust), which may be advantageous when changes to the plant occur (e.g., damage to the wings or added inertia of the body) and due to large external perturbations, such as a wind gust. It is worth noting that in flies, so-called optomotor saccades have a refractory period (Mongeau & Frye, 2017), which fundamentally limits the rate of switching and thus the ability of the saccadic system to keep the continuous system “in check”.

**REFERENCES**

**Bergou, A. J., Ristroph, L., Guckenheimer, J., Cohen, I. and Wang, Z. J.** (2010). Fruit Flies Modulate Passive Wing Pitching to Generate In-Flight Turns. *Phys Rev Lett* **104**, 148101.

**Cellini, B. and Mongeau, J.-M.** (2020). Hybrid visual control in fly flight: insights into gaze shift via saccades. *Curr Opin Insect Sci* **42**, 23–31.

**Cellini, B. and Mongeau, J.-M.** (2022). Nested mechanosensory feedback actively damps visually guided head movements in Drosophila. *Elife* **11**,.

**Cellini, B., Salem, W. and Mongeau, J.-M.** (2021). Mechanisms of punctuated vision in fly flight. *Current Biology* **31**, 4009-4024.e3.

**Cellini, B., Salem, W. and Mongeau, J.-M.** (2022). Complementary feedback control enables effective gaze stabilization in animals. *Proceedings of the National Academy of Sciences* **119**,.

**Chow, D. M. and Frye, M. A.** (2008). Context-dependent olfactory enhancement of optomotor flight control in Drosophila. *Journal of Experimental Biology* **211**, 2478–2485.

**Chow, D. M., Theobald, J. C. and Frye, M. A.** (2011). An Olfactory Circuit Increases the Fidelity of Visual Behavior. *The Journal of Neuroscience* **31**, 15035–15047.

**Dickinson, M. H. and Muijres, F. T.** (2016). The aerodynamics and control of free flight manoeuvres in *Drosophila*. *Philosophical Transactions of the Royal Society B: Biological Sciences* **371**, 20150388.

**Duistermars, B. J., Chow, D. M. and Frye, M. A.** (2009). Flies require bilateral sensory input to track odor gradients in flight. *Curr Biol* **19**, 1301–7.

**Elzinga, M. J., Dickson, W. B. and Dickinson, M. H.** (2012). The influence of sensory delay on the yaw dynamics of a flapping insect. *J R Soc Interface* **9**, 1685–1696.

**Fuller, S. B., Straw, A. D., Peek, M. Y., Murray, R. M. and Dickinson, M. H.** (2014). Flying *Drosophila* stabilize their vision-based velocity controller by sensing wind with their antennae. *Proceedings of the National Academy of Sciences* **111**, E1182–E1191.

**Mongeau, J.-M. and Frye, M. A.** (2017). *Drosophila* Spatiotemporally Integrates Visual Signals to Control Saccades. *Current Biology* **27**, 2901-2914.e2.

**Mronz, M. and Lehmann, F. O.** (2008). The free-flight response of *Drosophila* to motion of the visual environment. *Journal of Experimental Biology* **211**, 2026–2045.

**Rohrseitz, N. and Fry, S. N.** (2011). Behavioural system identification of visual flight speed control in Drosophila melanogaster. *J R Soc Interface* **8**, 171–185.

**Salem, W., Cellini, B., Kabutz, H., Hari Prasad, H. K., Cheng, B., Jayaram, K. and Mongeau, J.-M.** (2022). Flies trade off stability and performance via adaptive compensation to wing damage. *Sci Adv* **8**,.

**Salem, W., Cellini, B., Jaworski, E. and Mongeau, J.-M.** (2023). Flies adaptively control flight to compensate for added inertia. *Proceedings of the Royal Society B: Biological Sciences* **290**, 2022.12.15.520540.

**Wasserman, S., Lu, P., Aptekar, J. W. and Frye, M. A.** (2012). Flies dynamically anti-track, rather than ballistically escape, aversive odor during flight. *Journal of Experimental Biology* **215**, 2833–2840.

**Wasserman, S. M., Aptekar, J. W., Lu, P., Nguyen, J., Wang, A. L., Keles, M. F., Grygoruk, A., Krantz, D. E., Larsen, C. and Frye, M. A.** (2015). Olfactory Neuromodulation of Motion Vision Circuitry in Drosophila. *Current Biology* **25**, 467–472.
